# Agile workflow for interactive analysis of mass cytometry data

**DOI:** 10.1093/bioinformatics/btaa946

**Published:** 2020-12-14

**Authors:** Julia Casado, Oskari Lehtonen, Ville Rantanen, Katja Kaipio, Luca Pasquini, Antti Häkkinen, Elenora Petrucci, Johanna Hynninen, Sakari Hietanen, Olli Carpén, Mauro Biffoni, Anniina Färkkilä, Sampsa Hautaniemi

**Affiliations:** Research Program in Systems Oncology, Faculty of Medicine, University of Helsinki, Helsinki, Finland; Research Program in Systems Oncology, Faculty of Medicine, University of Helsinki, Helsinki, Finland; Research Program in Systems Oncology, Faculty of Medicine, University of Helsinki, Helsinki, Finland; Department of Pathology, University of Turku, Turku, Finland; Istituto Superiore di Sanità, Core Facilities, Rome, Italy; Research Program in Systems Oncology, Faculty of Medicine, University of Helsinki, Helsinki, Finland; Istituto Superiore di Sanità, Department of Haematology, Rome, Italy; Department of Obstetrics and Gynecology, Turku University Hospital, University of Turku, Turku, Finland; Department of Obstetrics and Gynecology, Turku University Hospital, University of Turku, Turku, Finland; Department of Pathology, University of Turku, Turku, Finland; Istituto Superiore di Sanità, Department of Haematology, Rome, Italy; Research Program in Systems Oncology, Faculty of Medicine, University of Helsinki, Helsinki, Finland; Department of Obstetrics and Gynecology, Helsinki University Hospital, Helsinki, Finland; Laboratory of Systems Pharmacology, Harvard Medical School and Dana-Farber Cancer Institute, Boston, MA, USA; Research Program in Systems Oncology, Faculty of Medicine, University of Helsinki, Helsinki, Finland

## Abstract

**Motivation:**

Single-cell proteomics technologies, such as mass cytometry, have enabled characterization of cell-to-cell variation and cell populations at a single-cell resolution. These large amounts of data, require dedicated, interactive tools for translating the data into knowledge.

**Results:**

We present a comprehensive, interactive method called *Cyto* to streamline analysis of large-scale cytometry data. *Cyto* is a workflow-based open-source solution that automates the use of state-of-the-art single-cell analysis methods with interactive visualization. We show the utility of *Cyto* by applying it to mass cytometry data from peripheral blood and high-grade serous ovarian cancer (HGSOC) samples. Our results show that Cyto is able to reliably capture the immune cell sub-populations from peripheral blood and cellular compositions of unique immune- and cancer cell subpopulations in HGSOC tumor and ascites samples.

**Availabilityand implementation:**

The method is available as a Docker container at https://hub.docker.com/r/anduril/cyto and the user guide and source code are available at https://bitbucket.org/anduril-dev/cyto.

**Supplementary information:**

Supplementary data are available at *Bioinformatics* online.

## 1 Introduction

Single-cell technologies, such as Cytometry by Time-Of-Flight (CyTOF), multiplexed imaging or single-cell RNA sequencing, have enabled characterizing tumor-microenvironment compositions and cell populations at a single-cell resolution (Galli *et al.*, 2019). However, currently the pace at which insight is extracted from massive single-cell datasets remains the same as with the previous low-throughput technologies (Brodin, 2019). Common CyTOF analysis steps have steadily reached a quasi-standard workflow that involves manual gating with FlowJo™ or other 2D scatter plot tools followed by dimensionality reduction with t-SNE (Van Der Maaten *et al.*, 2008) and unsupervised clustering. Typically, these analyses are executed with different software or platforms, which makes the results prone to errors and biases. Meanwhile, each new experiment requires a new set of custom scripts to fit the analysis needs, and new computational methods and algorithms are being developed at a fast rate (Angerer *et al.*, 2016; Höllt *et al.*, 2016; Qiu, 2017). The most comprehensive semiautomatic workflow available is CytoBank (Kotecha *et al.*, 2010), a commercially available service that allows the users to load the data to a cloud and perform analyses without the need for advanced technical skills. Also, open-source workflows with graphical interface, such as Cytofkit (Chen *et al.*, 2016), or command-line functions, such as cytofWorkflow (Nowicka *et al.*, 2017) have been published. These tools, however, integrate methods available within the R ecosystem where parallelization is not the default for most methods, which is a necessary feature to analyze very large datasets. Other, more complex solutions, such as Cytosplore (van Unen *et al.*, 2017) and CYT (Amir *et al.*, 2013), allow for only one method for each step of the analysis, one transformation type, one sampling approach, one clustering algorithm and one dimensionality reduction method. Furthermore, none of these software support iterative analysis, which is required for rapidly testing hypotheses and ideas during analysis. Iterative analysis is recognized as a key requirement for workflow languages (Almeida, 2010), and it is particularly important in the analysis of mass cytometry data as the datasets are complex and require testing different parameter settings, algorithms, etc. in an iterative and interactive fashion. We have designed and implemented an analysis software *Cyto* that enables interactive analysis and meets the need for accessibility to and reporting of reproducible methods.

We demonstrate the utility of *Cyto* with two CyTOF datasets. Firstly, we use control data from peripheral blood mononuclear cells (PBMC) (Van Unen *et al.*, 2016) to demonstrate fast quality assessment of the data and recapitulation of the previous findings in only two iterations of analysis. Secondly, we applied *Cyto* on a dataset from high-grade serous ovarian cancer (HGSOC). By applying *Cyto* on this dataset, we were able to rapidly measure abundance of cell types, and single-out specific tumor cell populations facilitating biological discovery and clinical interpretation of high dimensional single-cell cytometry data.

## 2 Materials and methods

Cyto is built on top of the workflow framework Anduril 2 (Cervera *et al.*, 2019), a language-agnostic framework that enables rapid integration of new and old methods as building blocks.

### 2.1 Cyto modules

#### 2.1.1 Graphical user interface

The user interface was developed as a light Flask application server within a Docker container. By distributing Cyto as an already built Docker image it avoids dependency installation and versioning issues, and therefore eases compatibility between researchers. The application handles data upload and download and saves user configuration changes. All projects are saved locally in the user’s computer in case Docker is restarted.

#### 2.1.2 Interactive results browser

To make *Cyto* modular, the user-data interaction was implemented as a separate web application built with Python dashboards, a powerful framework that supports interactive Plotly components. The choice of visualization strategies are based on those reported in relevant publications, particularly in Nowicka *et al.* (2017).

#### 2.1.3 Cytometry analysis pipeline

The analysis pipeline (Supplementary Fig. S1) is ran in the background. This means that, when the user clicks on ‘Run analysis’, Cyto will perform all steps of the analysis and parallelize when possible automatically. For ∼300 000 cells with default settings it takes less than 15 min on a standard MacBook Pro, for large datasets Cyto may run overnight unattended or in a computational environment with Docker support. Briefly, integration of cytometry specific methods was achieved through addition of new Anduril components built with MATLAB^®^, R, Python, Java or Bash scripts, depending on the programming language of the original implementation of each method. A list of the currently integrated methods for data processing, clustering, 2D embedding and descriptive statistics built into interactive dashboard elements are listed in Supplementary Table S1.

### 2.2 Materials and methods for peripheral blood case study

#### 2.2.1 Data acquisition of peripheral blood myeloid cells

We downloaded the mass cytometry FCS files from Van Unen *et al.* (2016) and selected the control (Ctrl) samples (*n *=* *14) to recapitulate the PBMC cell subtypes. No preprocessing of the data was required before the *Cyto* analysis.

#### 2.2.2 Cyto analysis of data quality

We selected the channels used in Van Unen *et al.* (2016). The complete dataset contained 48 611 486 cells, of which we randomly subsampled to 300 000 cells and transformed all selected channels with an arcsinh transformation (cofactor 5). The parameters and their values are listed in Supplementary File S1. Multidimensional scaling (MDS) and non-redundancy scores (NRS) visualization within *Cyto* Dash report were used to identify outlier samples.

#### 2.2.3 *Cyto* recapitulation of cell types

After excluding the outlier samples 52_CtrlAdult5_PBMC and 53_CtrlAdult6_PBMC we ran *Cyto* analysis (Supplementary File S2) on the remaining 12 Ctrl samples. This dataset contained 41 779 615 cells which were randomly downsampled to 300 000 cells. The same parameters as in the previous iteration were used but clustering was done with FlowSOM algorithm (*k *=* *18) and dimensionality reduction by tSNE (*n *=* *10 000; perplexity = 20; theta = 0.3). The cell type labels used and prior knowledge of marker expression profiles are described in Van Unen *et al.* (2016).

### 2.3 Materials and methods for case study HGSOC case study

#### 2.3.1 Data acquisition of high-grade serous ovarian cancer

Tissue and ascites specimens were collected from 15 consented patients (Supplementary Table S2) at the Department of Obstetrics and Gynecology, Turku University Central Hospital. Samples were analysed with CyTOF 1 mass cytometer (DVS Sciences Fluidigm). The antibody panel was manually curated with focus on markers of cell populations that compose the tumor compartment and less attention to the microenvironment (Supplementary Table S2). For further details about sample preparation and CyTOF assay see Supplementary Methods.

#### 2.3.2 Tumor compartment identification with *Cyto*

The FCS files and the CSV file with clinical annotations were uploaded to *Cyto* and processed as shown in Supplementary File S3. 300 000 cells were randomly sampled from a total of 65 331 333 cells in the complete dataset. After the cyto run with signal transformation *log1p*, sample-wise mean centering, clustering with Phenograph (*k *=* *200) and dimensionality reduction by UMAP (*n *=* *10 000, min-dist = 0.1, knn = 90). We associate cell types to each cluster based on the expression of canonical cell type markers (Supplementary Fig. S5). The clustering results were downloaded from *Cyto* to label the clusters and compare global cell type abundances. To maximize the number of tumor cells we ran a second iteration of analysis using a density-biased downsampling while keeping all other parameters unchanged (Supplementary Fig. S6). The resulting CSV file was filtered in AWK to keep only the tumor cells for the next iteration.

#### 2.3.3 Tumor cell population analysis

All tumor cells were used with no preprocessing (setting *none*). We applied all clustering methods to show the different effect of complex cell populations that do not follow a clear lineage on clustering results, each analysis is detailed with the method name within the configuration file Supplementary File S4.

## 3 Results

Cyto is an open-source application that enables running cytometry analysis pipelines that integrate state-of-the-art tools with reliable reporting and reproducibility as shown inFigure 1 (Dix *et al.*, 1998). Importantly, Cyto is designed to support key agile data analysis principles, for example, interactive visualization of the results with other scientists places focus on the individuals and takes it away from complex processes and plans. Saving and sharing Cyto configuration files supports systematic reporting and removes the need for ad hoc editing analysis script collections.

**Fig 1.**
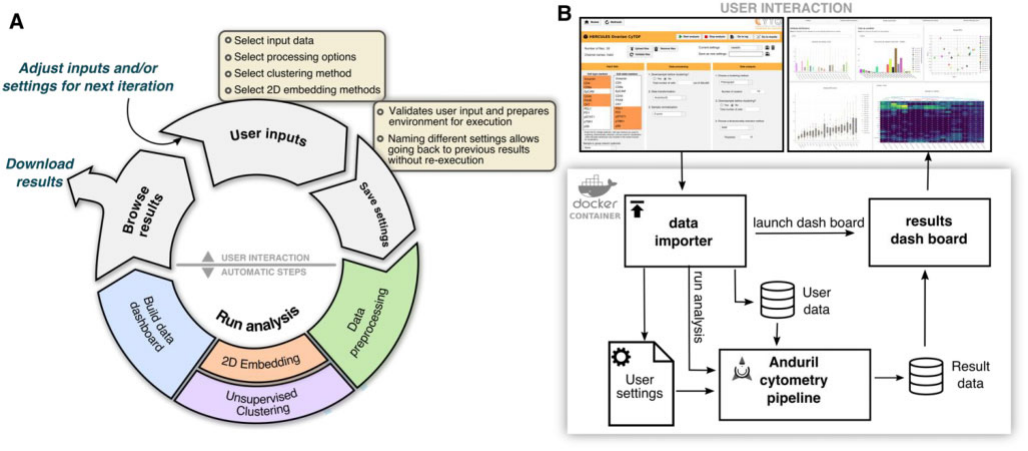
Workflow for cytometry analyses. (**A**) Diagram of steps showing cytometry analysis as an iterative process and how our framework enables knowledge discovery. (**B**) Schematic of the analysis environment to enable multi-system compatibility. On top screenshots of the *data importer* and the *results browser* as the two separate python applications

### 3.1 Software architecture supports reproducibility and accessibility requirements

The design of *Cyto* was driven by both the need of iterative analysis characteristic to the high dimensional cytometry field and the requirement for easily reporting of methods and parameters used in each step of an analysis, which are critical for reproducibility. With this in mind, we developed Anduril components to integrate the most popular cytometry tools into fully customizable analysis pipelines (https://bitbucket.org/anduril-dev/cytometry and https://bitbucket.org/anduril-dev/tools).

Our cytometry analysis pipeline includes tools from different fields and in different languages that are wrapped into modular units, called components, which are interchangeable and reusable throughout the pipeline development process. To enable rapid changes to the choice of components and to support non-bioinformaticians to interact with CyTOF datasets, we built a lightweight user interface that runs a generalizable Anduril pipeline (Supplementary Fig. S1). This is achieved with two web-based Python applications: the first one is the *data importer* where the user defines their analysis parameters, while the second one is the *results browser* to enable interactive data visualization through Plotly figures. Finally, to simplify installation requirements and thus enhance accessibility, we packaged this system into an interactive Docker container which can run on most operating systems. To our knowledge, *Cyto* is the first open-source solution that features access to multiple cytometry tools with a low learning threshold for non-bioinformaticians.

#### 3.1.1 Mass cytometry data analysis with Cyto

On a general scale, *Cyto* follows a common CyTOF workflow (Fig. 1A and Supplementary Fig. S1), however, each step enables agile and fast iterations. The *preprocessing* components are a critical step of a CyTOF pipeline. An *arcsinh* transformation is usually applied and it works well in many experiments, however, it may truncate high values to an artificial maximum. For this reason, users may choose also logarithmic or quadratic scaling. Other important parts of the preprocessing implemented in new components are quality assessment, normalization, gating and filtering components. By generalizing these steps in the *Cyto* pipeline instead of running multiple independent scripts or manual analysis, the user has a comprehensive log of methods tested, and complete control of the preprocessing steps without having to code all the logic that is already included in each component.

Because of the flexibility to adapt new tools as components to this bundle, *Cyto* supports *dimensionality reduction* and *unsupervised clustering* methods, along with new tools that can be included when available. The third popular toolbox contains *lineage inference* methods; we integrated them to produce an output that can be further analysed with any component or visualized with the *interactive visualization* components. The interactive visualization components transform data into plotly objects to be used either locally in the user’s browser or included in a Dash application, as demonstrated in the *Cyto* method. Lastly, Anduril counts with a large *tools* bundle with components for statistical analysis, CSV file manipulations and machine learning analysis, all of which are fully compatible with our cytometry components.

#### 3.1.2 Cyto design enables customized analysis steps

Worfklow for a standard cytometry analysis project is depicted in Figure 1A. First, the user sets the input data and parameters for the analysis in the *data importer*. Different types of research questions require different settings. Questions about population abundance can analyze all cells or a random sample, while detection and identification of rare cell populations requires a density-biased sample as implemented in SPADE package (Qiu *et al.*, 2011) to preserve smaller populations. Commonly used clustering algorithms in the field are tailored for different research setups (Weber et al., 2016). Algorithms based on a k-nearest neighbors approach are suitable for samples where the expression of markers varies smoothly, e.g., are expected to belong to an evolving. However, samples with distant subpopulations will benefit from a more fragmented clustering method, such as *k*-means. Thus, it is important to support the use of the right tool for the right question, not just the easiest to use. Second, the user saves the settings. At the moment of saving these options, *Cyto* validates the inputs and creates a new execution folder, which is used to archive the configuration, to support reproducibility, and to store the intermediate results, to support re-running only necessary steps on following iterations. Third, starting the analysis will launch the cytometry analysis pipeline and build the *results browser.* Upon completion of the analysis, the browser will enable the user to build new hypotheses and make informed decisions for the next iterations. The browser helps interacting with high-dimensional data and multiple results effectively, from assessing signal quality and sample selection quality to examining individual or groups of cell populations. In the *data importer*, we can also download the results as a table that includes all preprocessed data and clustering results, and the *results browser* can also be downloaded to be hosted on a web server as supporting material for complex publication results.

The presented cytometry components can also be integrated into Anduril pipelines independently of our proposed analysis pipeline within the *Cyto* system. Independent pipelines are specially useful for laboratories with highly specific research questions that cannot be addressed within the *Cyto* system but benefit from some of the steps. The modular design of our method enables other researchers to follow this design for specialized needs (Fig. 1B and Supplementary Fig. S1).

### 3.2 Case study I: peripheral blood myeloid cells dataset

#### 3.2.1 Interactive browser enables outlier detection

**Fig 2.**
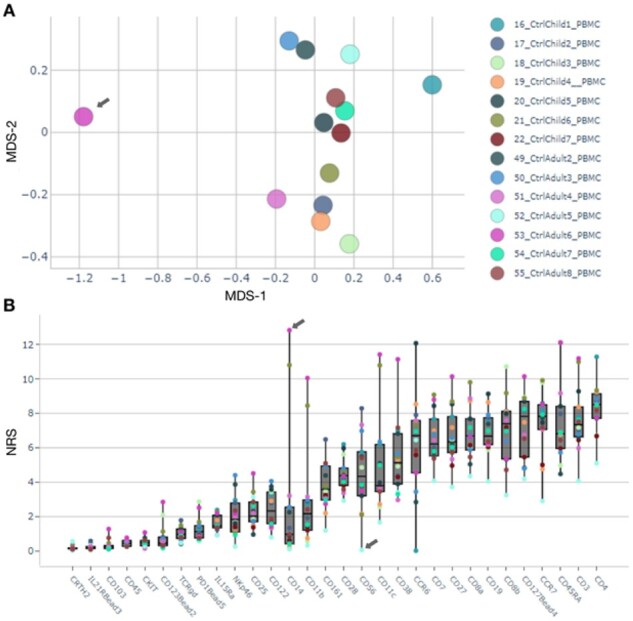
Outlier detection and characterization. (**A**) MDS plot shows sample 53_CtrlAdult6_PBMC separate from the other Ctrl samples. (**B**) Non-redundancy scores visualization; sample 53_CtrlAdult6_PBMC has highest NRS on marker CD14, and sample 52_CtrlAdult5_PBMC shows lowest for 18 out of 30 markers

The *results browser* generates summary figures to assess data quality. Multi-dimensional scaling visualization of the average expression on each sample (Fig. 2A) highlights sample *53_CtrlAdult6_*PBMC as an outlier at the general level. While visualization of Non-Redundancy Scores (Fig. 2B), identifies also sample *52_CtrlAdult5_PBMC* due to artifactually low signal, seen as lowest NRS for more than 50% of the antibodies. Further assessment of outlier samples is possible by exploring the profiles of cell populations predominant in the outlier population (Supplementary Fig. S2). In this analysis, sample *53_CtrlAdult6_PBMC* shows over-representation of myeloid cells, possibly caused by preanalytical conditions. Sample *52_CtrlAdult5_PBMC* shows a very low Simpson’s diversity index (0.34) compared with the rest of the samples (*µ*=0.67; *σ*=0.003) (Supplementary Fig. S3). By creating a new analysis from the *data importer*, we were able to rapidly discard poor quality samples and repeat the analysis with the same settings.

#### 3.2.2 Cyto recapitulates cell-type identification from PBMCs

**Fig 3.**
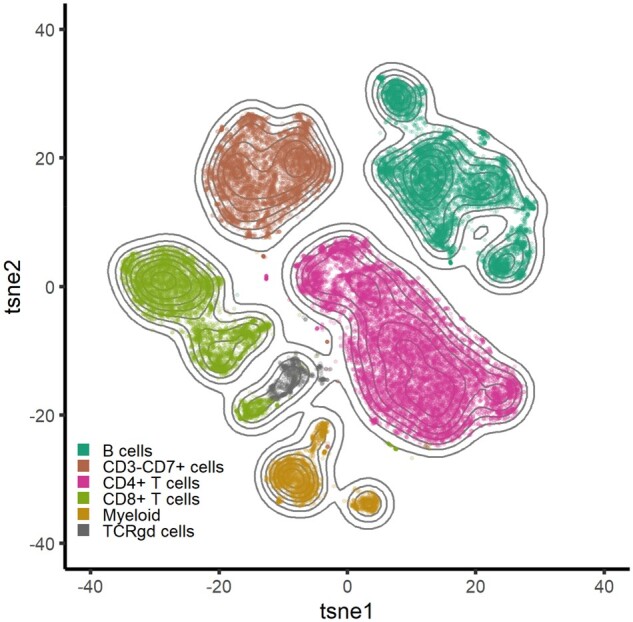
Recapitulation of cell types in the 12 PBMC samples using tSNE (*n* = 30 000, perplexity = 90, theta = 0.4) colored by the combined cluster labels produced by FlowSOM

We set out to test the performance of *Cyto* in detecting immune cell populations from the PBMC dataset. By using density-biased sampling, we quickly recapitulate the cell types present in these samples in line with the authors of the data. Figure 3 shows the results from *Cyto* manually colored by the cell type classification for each cell. Visual separation of some cell types can be further explored by intensity t-SNEs and lineage trees (Supplementary Fig. S4). Interactive visualization of relevant markers shows slight differences in expression within the same cell type. In addition, the lineages presented as the minimum spanning trees can be applied to the result of any clustering algorithm. *Cyto* analysis workflow herein reliably identifies biological cell populations from PBMC facilitating biological interpretation of CyTOF data.

### 3.3 Case study II: cancer cell populations on HGSOC

To assess the performance of *Cyto* in enabling clinical and CyTOF data integration we next performed an iterative analysis on a dataset of 15 clinical samples (Supplementary Table S2) from HGSOC patients at diagnosis (primary), after neoadjuvant chemotherapy (interval) or at tumor progression. In this analysis *Cyto* also takes advantage of a detailed clinical metadata to assist variable association in the *results browser*.

**Fig 4.**
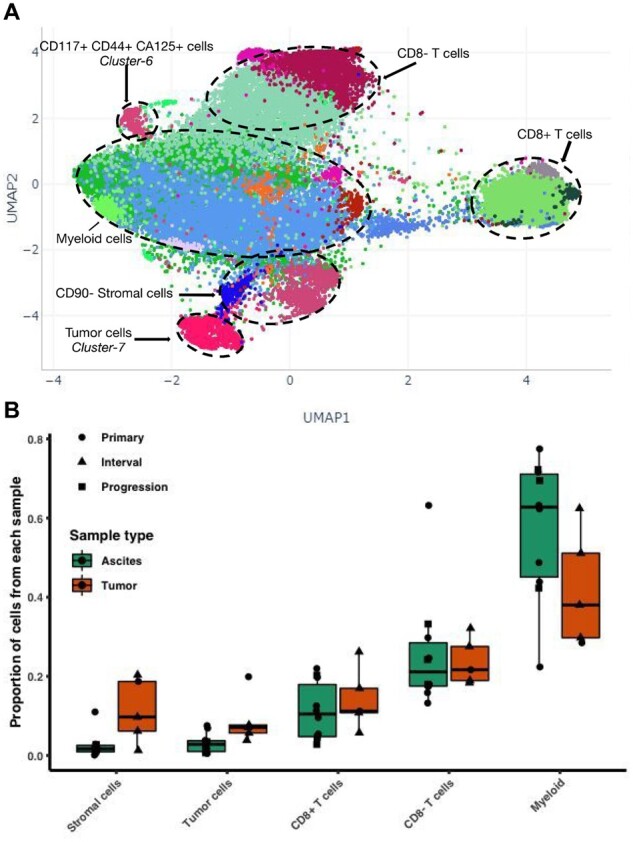
First iteration on high-grade serous ovarian cancer data. (**A**) Screenshot of all cells from 15 HGSOC samples from different therapy time-points and different tissue sites, Phenograph labels (colors) were computed with 300 000 cells randomly sampled and *k* = 450. (**B**) Summary of proportions of cell types identified by Phenograph for each sample annotated with sample type and tissue type

Phenograph successfully detects main immune, stromal and tumoral cells (Fig. 4A and Supplementary Fig. S5). The immune compartment is the largest; we annotated the clusters to be CD8+ T-cells, CD8- CD3+ likely CD4+T-cells and CD45+ T-cell marker negative likely Myeloid-lineage inflammatory cells. The stromal compartment is divided into CD90 positive and negative stromal cells, with the negative cells showing closer similarity to the tumor cells. The tumor compartment, identified as *Cluster-7* is characterized by high expression of EpCAM, MUC1, E-Cadherin and CA125, and low expression of pan-leucocyte marker CD45. Abundance difference (Fig. 4B) show ascites samples (*n* = 10) have more myeloid cells, and less tumor and stromal cells than solid tumor samples, while no apparent differences were observed on T-cell abundance. Interestingly, *Cluster-6* shows expression for stemness markers CD117 and CD44, the tumor markers CD125, HE4 and EpCAM, and is negative for the immune and stromal markers, presenting as a potential cancer stem cell population.

**Fig 5.**
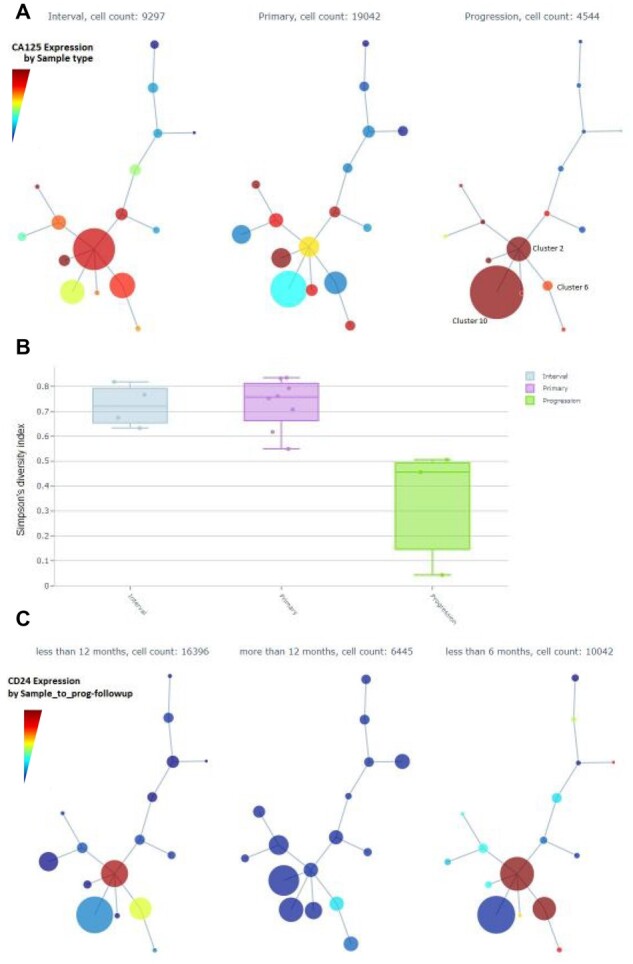
Screenshots of *Cyto* analysis of only tumor cell populations. (**A**) Minimum Spanning Trees (MST) by Sample time summarizes the expression of CA125. (**B**) Simpson’s diversity index by Sample time. (**C**) CD24 expression across MST nodes grouped by time from sample to next progression

A second iteration of Cyto analysis, in which we focused on the tumor cells (Fig. 5 and Supplementary Fig. S7), shows the integration of clinical annotations with a tumor subpopulation profiling analysis. The intermediate run that shows the detection of the tumor cells is shown in Supplementary Figure S6. Minimum spanning tree (MST) representation of the detected clusters present distinct tumor population abundance in Primary, Interval and Progression time of sampling. Furthermore, Cluster-6 on the MST shows higher Ki67 and more abundant in Primary and Interval samples. Cluster-2 shows highest E-Cadherin and is dominant in Interval samples, and progression samples have larger representation of Cluster-10, which are cells enriched for MUC1 and CD147, and are low on Ki67 and ERK1-2 signaling. A Cyto visualization of Simpson’s diversity index highlights also that Progression samples have the lowest heterogeneity.

Interestingly, collapsing the MST by time from sample to the next progression we see a clear enrichment of a stemness marker CD24 in samples with shorter time to progression. Detailed profiling of the tumor subpopulations can be explored in Supplementary Material.

## 4 Conclusion

Rapid advances in single-cell technologies produce larger and more complex data than ever before. The need for several analyses increase the difficulty of reporting reproducible results, while accessibility to and usability of highly specialized tools drive the choice of algorithms in the analysis. A standard one-way analysis workflow is sufficient on low-dimensional data but a more exploratory research requires an iterative approach. We propose to level the usability of different tools and to ease reproducibility of analysis by integrating tools using a workflow paradigm design. First, by including popular cytometry methods as Anduril components available, less experienced bioinformaticians can easily build customized analysis workflows. Second, we present a generalized analysis pipeline that covers cytometry questions from detection of rare cells to differential abundance analysis, and from general sample profiling to deeper analysis of single cell populations. Third, by making this pipeline accessible as a Docker container with a user-friendly interface, non-bioinformaticians are able to perform complex single-cell analyses regardless of their experience level on software maintenance. Fourth, a side-effect of utilizing Docker for accessibility includes the potential to run it remotely on a server.

To our knowledge, *Cyto* is the first cytometry tool with a workflow paradigm design. Many R packages (Finak *et al.*, 2014; Simpson, 2019; Spidlen *et al.*, 2019) have enabled compatibility with the popular flowCore package (Ellis *et al.*, 2019), and including them in our cytometry components allow users to execute them as part of larger pipelines on computing clusters if necessary. Future integration of new cytometry tools was supported by Anduril syntax as wrappers in the form of new components, components can be written in the user’s language of choice due to Anduril’s language-agnostic design.

In addition, this study demonstrates the key features of Cyto on a public, well-known dataset, as well as on a new independent cohort. Here we are able to identify and characterize cell population changes before and after chemotherapy, as well as at the time of progression. Ascites samples are valuable but underutilized due to the large number of non-tumor cells. Our analysis characterized the composition of the herein used ascites samples and the iterative analysis feature in Cyto enabled focusing on tumor cells without manual setting of thresholds for each channel and sample. This allowed us to compare tumor cell phenotypes between clinical presentations, suggesting that HGSOC tumors at relapse phase are characterized by lower heterogeneity while those with shorter time to next progression showed enriched stemness; both yield potential hypotheses for further studies.

In summary, this work presents Cyto, which is an open-source, accessible and customizable cytometry analysis method that takes advantage of workflow engines and enables easy integration of existing tools. Cyto offers two levels for technical and non-technical users. Further, to our knowledge this study presents the first CyTOF experiments on comparison of chemotherapy naïve and heavily treated relapse samples from HGSOC.

## Supplementary Material

btaa946_Supplementary_Data
