# Supplementary material for: Agile workflow for interactive analysis of mass cytometry data
Source: Bioinformatics. 2020 Dec 14;37(9):1263–8. doi: 10.1093/bioinformatics/btaa946 (PMC8189671; doi:10.1093/bioinformatics/btaa946)
Supplement: btaa946_Supplementary_Data [file btaa946_supplementary_data.zip › casado_et_al_Supplementary.docx]

Pages xxx-xxx

| Systems Biology  **Supplementary Material**  **Julia Casado^1^, Oskari Lehtonen^1^, Ville Rantanen^1^, Katja Kaipio^2^, Luca Pasquini^3^, Antti Häkkinen^1^, Elenora Petrucci^4^, Johanna Hynninen^5^, Sakari Hietanen^5^, Olli Carpén^2^, Mauro Biffoni^4^, Anniina Färkkilä^1,6,7^, and Sampsa Hautaniemi^1,*^**  ^1^Research Program in Systems Oncology, University of Helsinki, Finland; ^2^Department of pathology, University of Turku, Finland; ^3^Istituto Superiore di Sanità, Core Facilities, Rome, Italy; ^4^Istituto Superiore di Sanità, Department of Haematology, Rome, Italy; ^5^Department of Obstetrics and Gynecology, Turku University Hospital, University of Turku, Turku, Finland; ^6^Department of Obstetrics and Gynecology, Helsinki University Hospital; ^7^Dana-Farber Cancer Institute, Harvard Medical School, Boston, MA;  *To whom correspondence should be addressed. |
| --- |

[1 Supplementary methods 1](#_Toc41600172)

[1.1 Data acquisition of High-Grade Serous Ovarian Cancer 1](#_Toc41600173)

[1.1.1 Sample dissociation and preparation 1](#_Toc41600174)

[1.1.2 Antibody preparation for mass cytometry 1](#_Toc41600175)

[1.1.3 Cell processing and antibody staining 2](#_Toc41600176)

[1.1.4 CyTOF assay and data preprocessing 2](#_Toc41600177)

[2 Supplementary tables 3](#_Toc41600178)

[3 Supplementary figures 5](#_Toc41600179)

# Supplementary methods

## Data acquisition of High-Grade Serous Ovarian Cancer

### Sample dissociation and preparation

Ovarian cancer primary cells were isolated from ascites and tumor tissues. Ascites was centrifuged at 3.0 G for 15 min, followed by gradient centrifugation with Histopaque-1077 to discard the contaminating blood cells from the sample. Tissues were cut in to approximately 1mm pieces and dissociated over night with 1:75 dilution of 10x Collagenase/hyaluronidase in warm DMEM-F12 media (Stem Cell technologies, Cambridge, UK). Cells were isolated by filtering the sample with 100μm and 70 μm meshes followed by Histopaque-1077 centrifugation to discard contaminating blood cells and cell debris.

### Antibody preparation for mass cytometry

The antibodies (Supplementary Table S2) were purchased already conjugated with metal isotopes from Fluidigm when available. Otherwise, purified carrier-free antibodies were purchased from other vendors (Biolegend, R&D System and BD Biosciences) and then conjugated with metal isotopes using the Maxpar antibody conjugation kit (Fluidigm) following the manufacturer’s instructions. In-house conjugated antibodies were quantified and diluted in PBS antibody stabilization solution (CANDOR Biosciences) to 0.1-0.4 mg/ml and stored at +4°C. CD166, CD133 and cleaved-PARP were purchased conjugated with fluorochromes and detected with anti-fluorochromes metal tagged antibodies (145Nd-PE, 176Yb-APC and 160Gd-FITC respectively) in a secondary staining step. Antibodies have been initially tested by flow cytometry followed by a titration at mass cytometer using cell lines to set the working concentration.

### Cell processing and antibody staining

The isolated cells were washed with 1x PBS, centrifuged, suspended in to warm DMEM-F12 medium and stained with 1 μM 103Rhodium- DNA Intercalator (Fluidigm). Unstained cells were acquired as control sample to detect the background signals. After 15 min incubation at 37°C, cells were washed with Cell Staining Medium (CSM) [PBS, 0.5% BSA (Sigma Aldrich), 0.02 % NaN3 (Sigma Aldrich)] and fixed with 1.6% paraformaldehyde (Electron Microscopy Sciences) for 10 min at room temperature. Samples were washed twice with CSM and shipped at +4°C to the Istituto Superiore di Sanità, Roma, Italy. Upon arrival the cells were counted and around 2-3 million were pelleted, washed with CSM and incubated with Fc-blocker (Biolegend) for 10 minutes at RT to counteract the antibody binding to FC-receptors. Cellular staining with antibodies was performed according to the manufacturer’s protocol (Fluidigm) consisting of several staining steps interspersed with permeabilization treatments moving from gentlest to strongest conditions. In the first step, cells were resuspended in 100 μl of a mix of CSM and metal-conjugated antibodies specific for surface antigens, incubated for 30 minutes at RT and washed twice in CSM. In a second step the cells were permeabilized with 1ml of CSM supplemented with 0.3% Saponin (Sigma Aldrich) (CSM-S) for 30 minutes at +4°C and then stained with an antibody cocktail specific for intracellular antigens, for 45 minutes at RT and washed twice with CSM-S. The third step consisted in a stronger permeabilization of the cell pellet with 1 ml of ice cold methanol (Sigma Aldrich) per 0.5x10^6^ cells for 10 minutes at 4°C. Cells were then washed twice in CSM and stained with 100 μl of a further antibody mix toward phosphoproteins and transcription factors, for 60 minutes at RT in CSM. After washing with CSM, the cells were stained with 125 nM 191/193Iridium-DNA Intercalator (Fluidigm), in PBS/PFA 1.6% for 20 minutes at RT (or overnight at 4), for cell events recognition during data acquisition, and then washed twice with CSM and once with MilliQ water.

### CyTOF assay and data preprocessing

Before acquisition, cells were counted and diluted at 2x10^5^ cells/ml in MilliQ water with 1/10 of volume of EQ^TM^ Four Element Calibration Beads (Fluidigm) and filtered through a 35μm nylon mesh before acquisition. Data from each sample were pre-processed with CyTOF software version 6.7.1014 to normalize signals and minimize instrument performance variation during acquisition (lower convolution threshold of 200, event length between 10 and 75 and with a rate of 500 cells/sec). FCS files were processed with FlowJo software (FlowJo LLC) to export bead-normalized single-viable cells based on gating performed on cell length and DNA intercalators signals (191/193Iridium and 103Rhodium). Because each sample was processed at the time of acquisition to conserve signal quality, the header of the raw FCS files were matched in R.

# Supplementary tables

**Table S1.** Methods and tools available within the Anduril pipeline integrated in *Cyto*.

| **Category** | **Method/Tool** |
| --- | --- |
| Preprocessing | Density-biased sampling |
|  | Random down sampling |
|  | Data tranformation |
|  | Sample-wise normalization |
| Clustering | Phenograph |
|  | FlowSOM |
|  | FlowMeans |
|  | XShift |
|  | K-means |
| 2D Embedding | tSNE |
|  | UMAP |
| Build Data Dashboard | Multidymensional scalling |
|  | Distance correlation |
|  | Summary statistics |
|  | Non-redundancy score |
|  | Minimum Spanning trees |

**Table S3.** Sample cohort included in this study. Age, stage, tissue, number of patients, number of total cells acquired from each, survival, treatment.

| **Sample file** | **Patient code** | **Age at diagnosis** | **Histological grade** | **Treatment phase** | **Tissue site** | **PFI months** | **OS months** |
| --- | --- | --- | --- | --- | --- | --- | --- |
| EOC1_r2Asc.fcs | EOC1 | 71 | IIIC | Recurrence | Ascites | 6 | 21.9 |
| EOC2_rAsc2.fcs | EOC2 | 39 | IVB | Recurrence | Ascites | 13.1 | 31.86 |
| EOC3_pAsc.fcs | EOC3 | 77 | IVB | Primary | Ascites | 16.79 | 34.7 |
| EOC4_iAsc.fcs | EOC4 | 60 | IIIC | Interval | Ascites | 2.1 | >38 |
| EOC4_pAsc.fcs |  |  |  | Primary | Ascites |  |  |
| EOC5_iOme.fcs | EOC5 | 67 | IIIC | Interval | Omentum | 9.2 | 30.76 |
| EOC6_pAsc2.fcs | EOC6 | 68 | IVB | Interval | Ascites | 2.3 | 14.63 |
| EOC7_iAsc.fcs | EOC7 | 75 | IIIC | Interval | Ascites | 6.9 | >38 |
| EOC7_iMes.fcs |  |  |  | Interval | Mesentery |  |  |
| EOC7_pAsc.fcs |  |  |  | Primary | Ascites |  |  |
| EOC8_pAsc.fcs | EOC8 | 54 | IVA | Primary | Ascites | 5.8 | 29.7 |
| EOC8_pOme.fcs |  |  |  | Primary | Omentum |  |  |
| EOC9_pAsc.fcs | EOC9 | 62 | IIIC | Primary | Ascites | 3.5 | 17.9 |
| EOC10_iOme.fcs | EOC10 | 72 | IVA | Interval | Omentum | 2.7 | 19.17 |
| EOC11_iOme.fcs | EOC11 | 73 | IVA | Interval | Omentum | 2.1 | >25 |
| EOC12_pAsc.fcs | EOC12 | 78 | IVA | Primary | Ascites | 12.9 | >24 |
| EOC13_pAsc.fcs | EOC13 | 60 | IIIC | Primary | Ascites | >19 | >24 |
| EOC14_pAsc.fcs | EOC14 | 67 | IIIC | Primary | Ascites | 9.6 | >22 |
| EOC15_r2Asc.fcs | EOC15 | 64 | IIIC | Recurrence | Ascites | 5.7 | 28.17 |

**Table S2.** Antibodies used for in-house CyTOF data.

| ***Cat No*** | ***Metal Tag*** | ***Target*** | ***Clone*** | ***Vendor*** | ***Final Concentration (μg/100μl)*** | ***Dilution*** |
| --- | --- | --- | --- | --- | --- | --- |
| 3141006B | 141Pr | EpCAM | 9C4 | Fluidigm | N.A | 1:50 |
| 3143001B | 143Nd | CD117 | 104D2 | Fluidigm | N.A | 1:50 |
| MAB56091 | 144Nd | CA125 | 986811 | R&D | 1.2 | - |
| 559263/3145006B | 145Nd | CD166-PE | 3A6/PE001 | BD/Fluidigm | N.A | 1:30/1:50 |
| 3147015B | 147Sm | ALDH | 44/ALDH | Fluidigm | N.A | 1:50 |
| 304045 | 149Sm | CD45 | HI30 | Biolegend | 0,6 | - |
| 561469 | 151Eu | Sox2 | O30-678 | BD | 1.2 | - |
| 3152005A | 152Sm | pAkt [S473] | D9E | Fluidigm | N.A | 1:50 |
| 3153021B | 153Eu | CD44s Pan Specific | 691534 | Fluidigm | N.A | 1:75 |
| 3154003B | 154Sm | CD3 | UCHT1 | Fluidigm | N.A | 1:75 |
| 355602 | 155Gd | MUC1 | 16A | Biolegend | 0.7 | - |
| 3156022B | 156Gd | CD147 | HIM6 | Fluidigm | N.A | 1:75 |
| 3158021A | 158Gd | E-Cadherin | 24E10 | Fluidigm | N.A | 1:50 |
| 3159029B | 159Tb | PD-L1 | 29E.2A3 | Fluidigm | N.A | 1:50 |
| 558576/3160011B | 160Gd | Cleaved PARP-FITC | F21-852/FIT22 | BD/Fluidigm | N.A | 1:30-1:50 |
| 3161009B | 161Dy | CD90 | 5E10 | Fluidigm | N.A | 1:75 |
| 3162015B | 162Dy | CD8a | RPA-T8 | Fluidigm | N.A | 1:75 |
| MAB6274 | 164Dy | HE4 | 676013 | R&D | 0.9 | - |
| 350802 | 165Ho | N-Cadherin | 8C11 | Biolegend | 0.9 | - |
| 550314 | 166Er | CD146 | P1H12 | BD | 1.2 | - |
| 3171010A | 167Er | pERK 1/2 [T202/Y204] | D13.14.4E | Fluidigm | N.A | 1:50 |
| 3168007B | 168Er | Ki-67 | B56 | Fluidigm | N.A | 1:50 |
| 3169004B | 169Tm | CD24 | ML5 | Fluidigm | N.A | 1:50 |
| 3172014B | 172Yb | PD-L2 | 24F.10C12 | Fluidigm | N.A | 1:50 |
| 3174020B | 174Yb | PD-1 | EH12.2H7 | Fluidigm | N.A | 1:50 |
| 3175009A | 175Lu | pS6 [S235/S236] | N7-548 | Fluidigm | N.A | 1:50 |
| 130-090-826/3176007B | 176Yb | CD133-APC | AC133/APC003 | Miltenyi/Fluidigm | N.A | 1:30-1:50 |

#
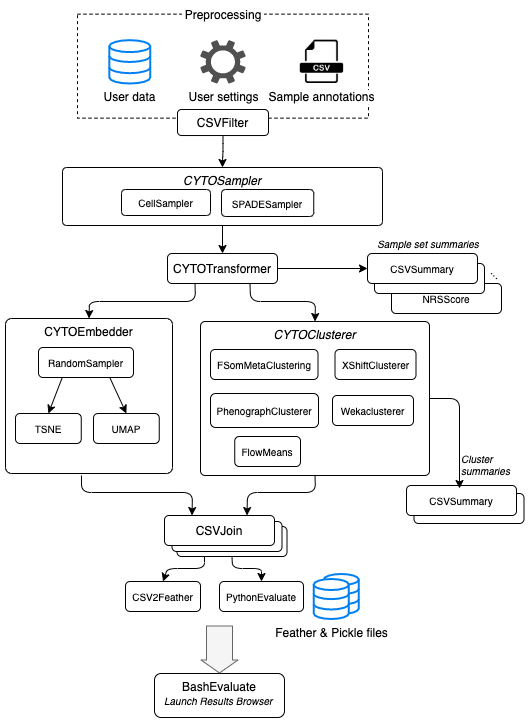
Supplementary figures

**Figure S1.** Detailed workflow of the Anduril workflow used in *Cyto*.


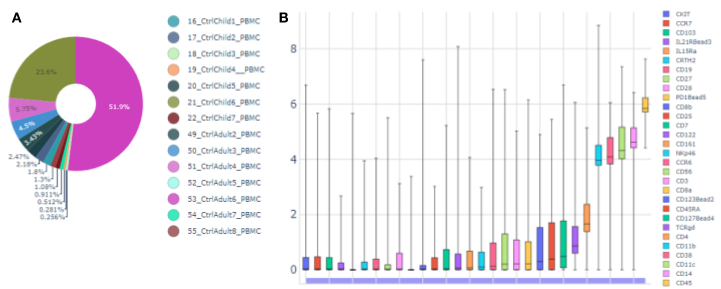


**Figure S2.** (A) Sample representation within the dominant cluster from the outlier sample 53_CtrlAdul6_PBMC. (B) Marker signal distribution within this cluster. The highest expressing markers are CD14, CD11c, CD38, and CD11b.

**
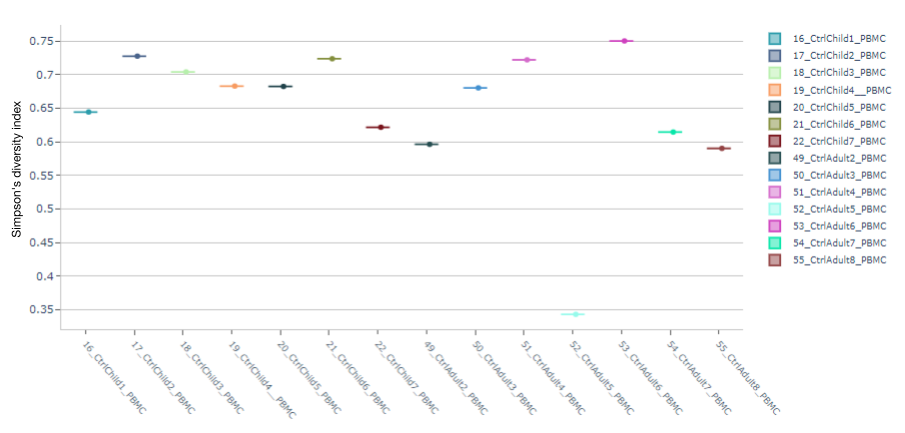
**

**Figure S3.** Simpson’s diversity index identifies sample 52_CtrlAdult5_PBMC (in Cyan) as an outlier based on the number of clusters represented within the sample and the relative abundance of each of the clusters.

**
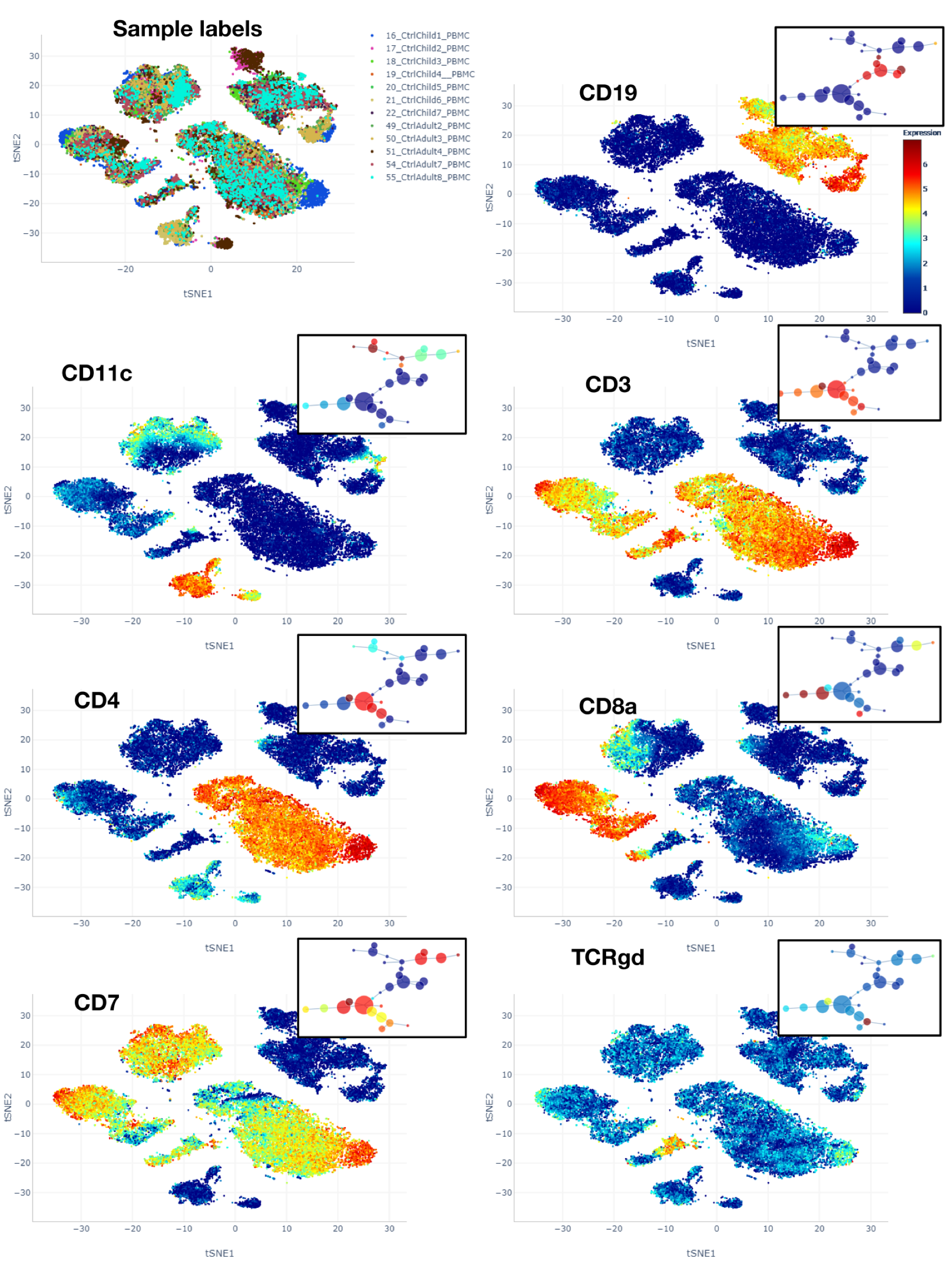
**

**Figure S4.** Expression profiles of the PBMC cell types without the two outlier samples. The sample labels indicate that a batch effect is not dominant. Visualization of the dataset as tSNE or as MST shows the expression and relationships of the populations shown in Figure 3.

**
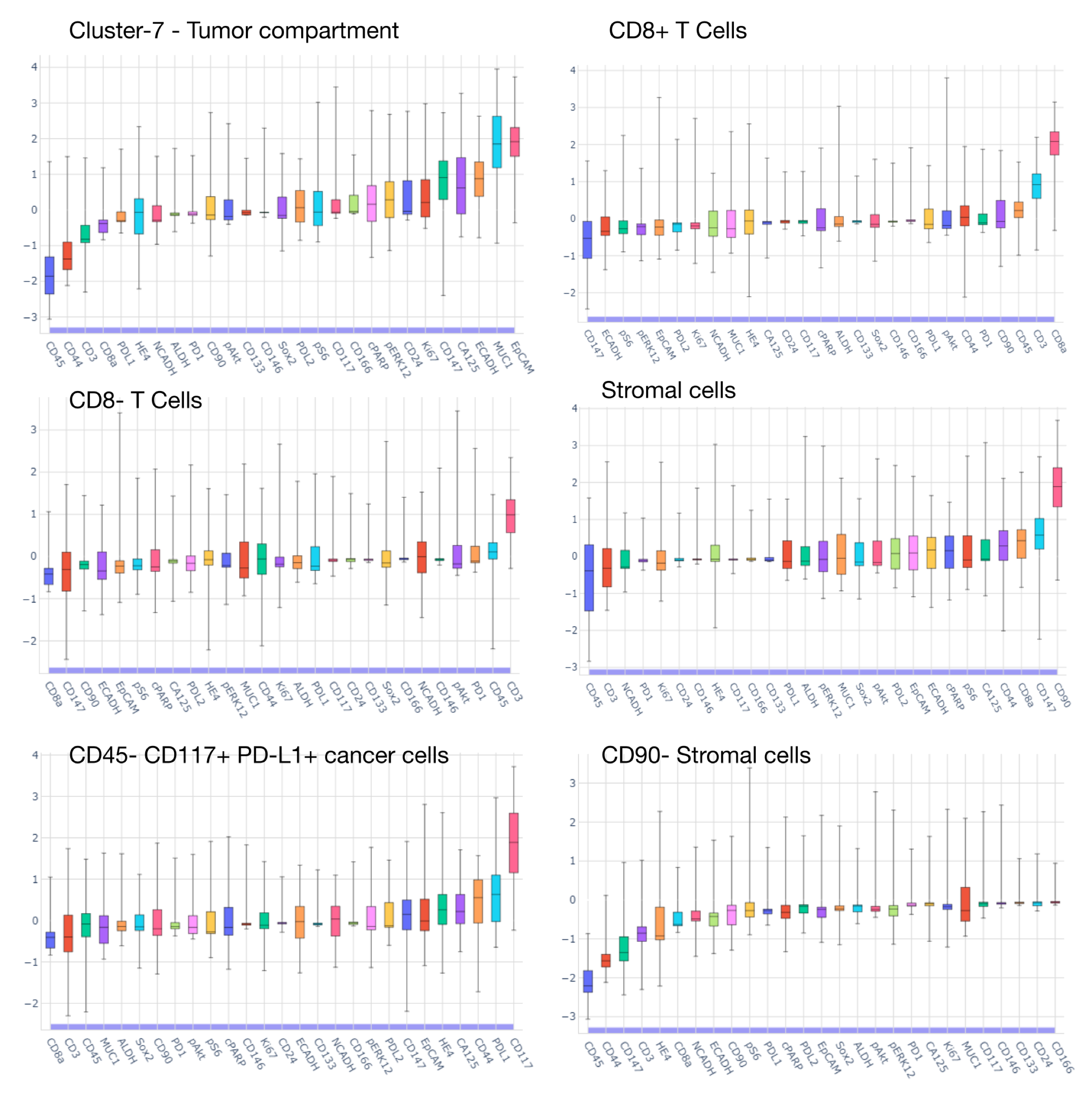
**

**Figure S5.** Expression distribution of each marker within the cell clusters selected in Figure 4. The lasso selection tool in the results browser allows to explore the expression profiles interactively.

**Figure S6.** Identification of the tumor compartment using density-based sampling option. (A) UMAP of the density-based sampled dataset with the tumor cells highlighted with a dashed line. (B) Expression profile of each cluster. Tumor cells are highlighted with a dashed line and further filtered for the tumor cell analysis.


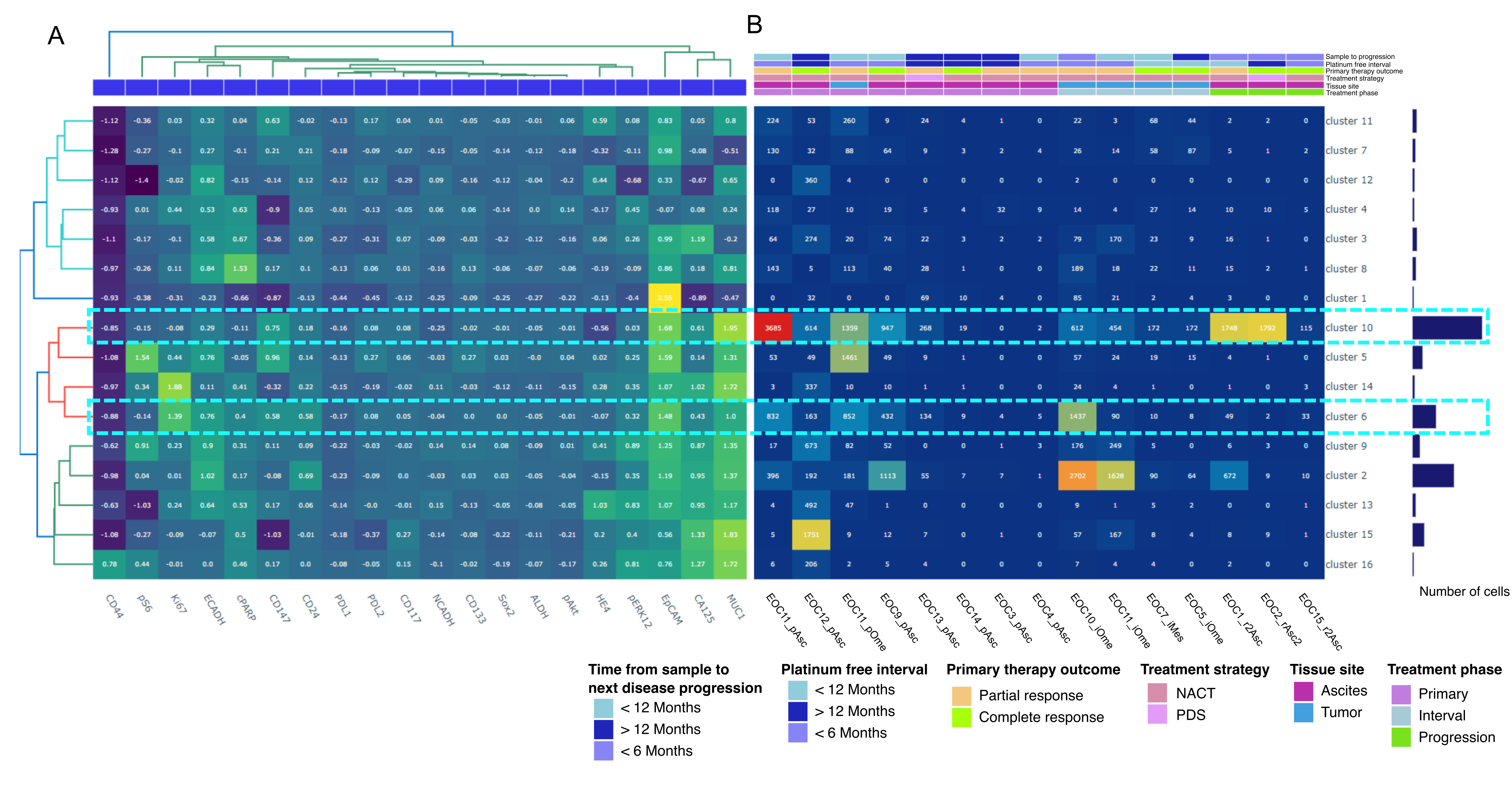

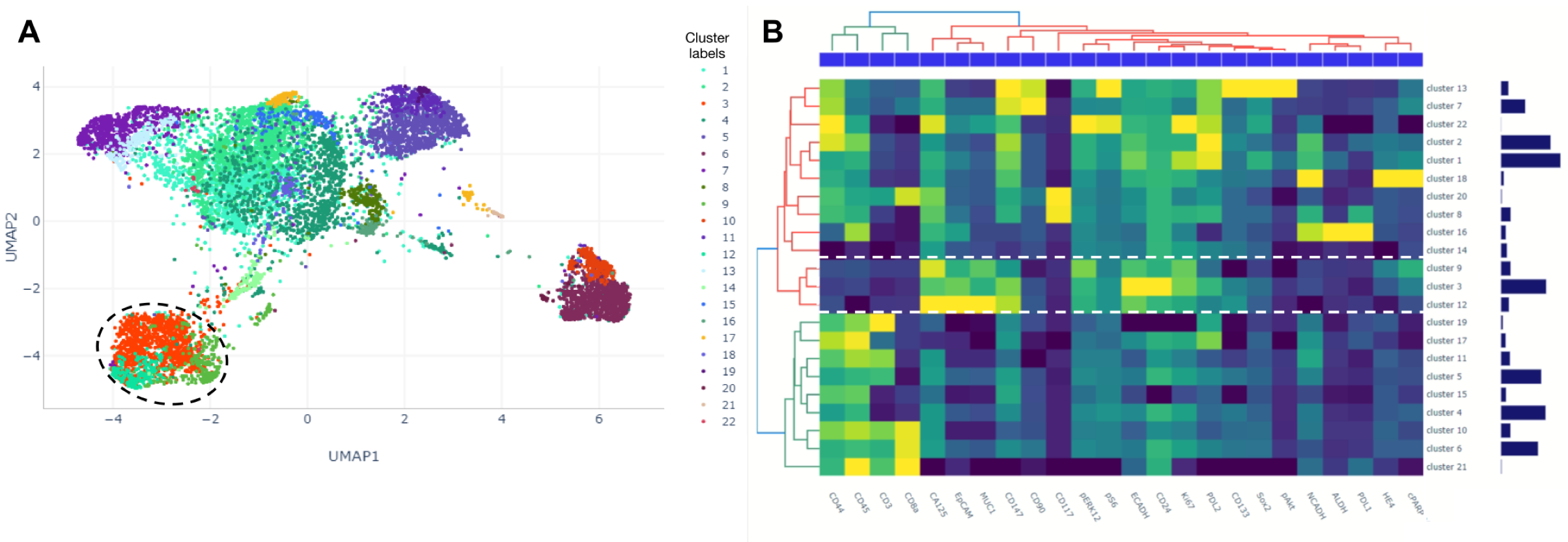


**Figure S7.** Tumor cell clustering with focus on Cluster-10 and Cluster-6 (highlighted in cyan) (A) Marker expression and hierarchical clustering of the subpopulations. (B) Population abundance across the samples.
